# Diagnosis and management of unilateral subclavian steal syndrome with bilateral carotid artery stenosis

**DOI:** 10.1016/j.amsu.2021.102597

**Published:** 2021-07-26

**Authors:** David Song, Branden Ireifej, Tasur Seen, Talal Almas, Yasar Sattar, M. Chadi Alraies

**Affiliations:** a Icahn School of Medicine at Mount Sinai Elmhurst Hospital, Queens, NY, USA; b Royal College of Surgeons in Ireland, Dublin, Ireland; c Division of Interventional Cardiology, Detroit Medical Center, Detroit, MI, USA

**Keywords:** Subclavian steal syndrome, Vertebral artery stenosis, Carotid stenosis. endovascular

## Abstract

Subclavian steal syndrome is a rare phenomenon occurring from retrograde blood flow in the vertebral artery due to proximal stenosis in the subclavian artery. As a result, the arm gets blood supply from the vertebral artery at the expense of the vertebrobasilar system. The patient remains largely asymptomatic until there is an increase demand for blood supply to the arm, resulting in a constellation of symptoms including dizziness, vertigo, blurred vision, diplopia, headache, syncope, postural hypotension, neurologic deficits, and rarely, memory problems. The management approach depends on the severity of clinical symptoms but includes medical treatment, endovascular therapy and lifestyle modifications.

## Highlights

• Subclavian steal syndrome with concurrent carotid artery stenosis is only the second documented case, which makes it an extremely rare presentation or frequently unnoticed condition.
• Absence of symptoms does not rule out subclavian steal syndrome with bilateral carotid stenosis and should be considered in the differential in patients with high risk comorbidities with appropriate imaging and physical exam.
• The treatment depends on the severity of clinical symptoms but includes medical treatment, endovascular therapy and lifestyle modifications.

## 1 Introduction

Subclavian steal syndrome is defined as the reversal of blood flow due to significant stenosis of proximal subclavian artery. This syndrome was first described in 1960 by Contorni [1]. The patient can be symptomatic or asymptomatic depending on the degree of stenosis. The symptoms vary from upper extremity claudication, vertigo, diplopia or syncope. A blood pressure difference of >15 mmHg in upper arms is suggestive of the subclavian stenosis; therefore a thorough investigation by ultrasound doppler, magnetic resonance angiogram (MRA) and computed tomography angiography (CTA) is conducted to delineate the diagnosis [1]. The management approach depends on the severity of clinical symptoms but includes medical treatment, endovascular therapy and lifestyle modifications.

## 2 Case presentation

A 55 year old male with an 80 pack year smoking history presented to the emergency department with left sided occipito-parietal headache, vertigo, ataxia, nausea, intermittent claudication of the left extremity and recurrent hypotension in the left upper extremity. He reported recurrent symptoms of syncope in the last four months. He had been admitted multiple times with orthostatic hypotension and intermittent posterior neurological signs. His past medical history includes T-Cell Lymphoma in remission and left frontal stroke with prior carotid artery stenosis. At this visit, the patient denied fever, chills, chest pain, shortness of breath, or any focal neurological deficits. At the presentation, he had blood pressure (BP) variation between right and left upper extremity. The BP in the left upper extremity was 80/65 mmHg and 110/85 mmHg in the right upper extremity yielding an upper extremity systolic BP difference of greater than 20 mmHg. The neurological examination showed intermittent pain on flexion of the left upper extremity, ataxia, vertigo, memory deficit and diplopia on walking. On further examination, a bilateral carotid and left subclavian bruit were noted. In addition, this work has been reported in accordance with SCARE [2].

### 2.1 Investigations

**Fig. 1:**
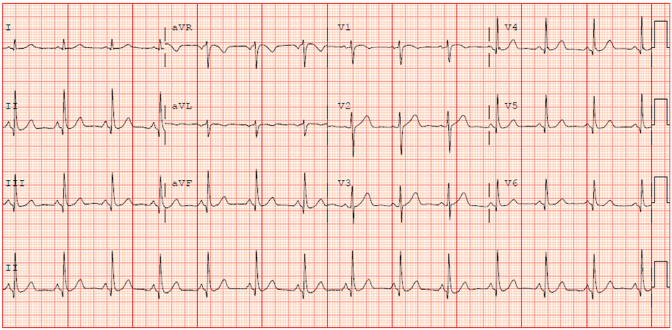
The ECG shows normal sinus rhythm with slightly short PR interval and minimal ST depression in inferior leads.

**Fig. 2:**
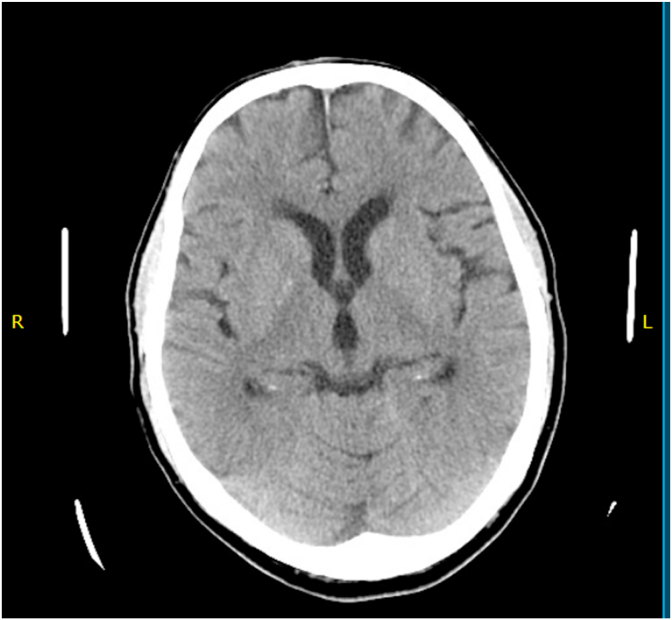
The horizontal section of the brain shows no evidence of acute intracranial pathology.

**Fig. 3:**
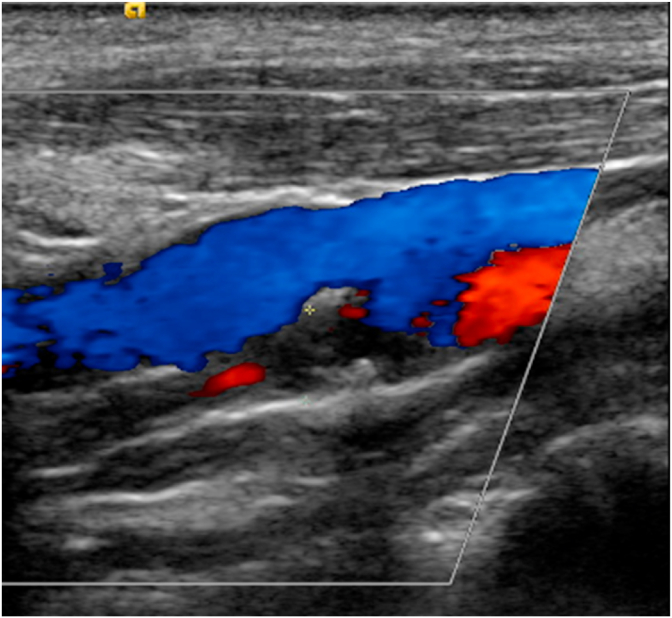
This figure illustrates the color Doppler image showing reversal of blood flow. 50% stenosis of the right carotid artery bulb due to extensive fibrofatty plaque with a transluminal flow of 0.425 m/s in the right internal carotid and right subclavian artery. (For interpretation of the references to color in this figure legend, the reader is referred to the Web version of this article.)

The baseline electrocardiogram showed normal sinus rhythm [Fig. 1] and chest radiograph was normal. The head CT scan at the presentation showed chronic left frontal lobe infarct otherwise unremarkable [Fig. 2]. Baseline laboratory examination showed hyperglycemia at 143 mg/dL (normal range 70–140 mg/dL) and cholesterol levels 109 mg/dL (normal less than 200 mg/dL), LDL-cholesterol 46 mg/dL (normal less than 100 mg/dL) and HDL-Cholesterol of 51 mg/dL (normal range 40–60 mg/dL). The rest of the laboratory examination was normal including Troponin-T, coagulation studies, electrolyte panel and complete blood count. The echocardiogram demonstrated a 60% ejection fraction with normal chamber sizes and trace mitral and tricuspid regurgitation. The carotid duplex revealed 99% stenosis of the left carotid artery bulb with extensive fibrofatty plaque and only small transluminal flow of 0.34 m/s across the left internal carotid artery. The left vertebral artery showed reversal of the flow, suggestive of subclavian steal syndrome. There was a 50% stenosis of the right carotid artery bulb due to extensive fibrofatty plaque with a transluminal flow of 0.425 m/s in the right internal carotid and right subclavian artery [Fig. 3].

### 2.2 Treatment

Owing to the concern of unilateral occipito-parietal headache, the patient initially received intravenous toradol 15 mg and zofran 4 mg. In the light of the diagnostic findings of carotid duplex, differential blood pressure in the upper extremity and clinical symptoms, a diagnosis of left subclavian steal syndrome with bilateral carotid artery stenosis was made.

The department of neurosurgery was consulted to assess the patient for the possibility of endovascular procedure. Patient was subsequently scheduled for left carotid endarterectomy and left subclavian angioplasty. At discharge, the patient was counselled for lifestyle changes, strict smoking cessation, strict glycemic control, and antithrombotic therapy to prevent restenosis. The patient had a positive outcome after the procedure with resolution of symptoms. Thereafter, he continued the lifestyle changes for the secondary prevention of restenosis.

## 3 Discussion

Subclavian steal syndrome is a manifestation of subclavian artery occlusion that is precipitated by atherosclerosis or vasculitic lesions [3]. This compromises the perfusion to the internal mammary and axillary artery. It may present as an upper arm claudication or vertebrobasilar hypo-perfusion resulting in vertigo, diplopia and syncope [4]. The prevalence in the general population is around 3–4% [4] with most patients being asymptomatic and only a small proportion (5%) presenting with symptoms. The condition usually presents in the context of cardiovascular risk factors such as hypertension, hyperlipidemia, and smoking history.

The clinical suspicion is raised by a discordant blood pressure (>15 mmHg difference in systolic BP), delayed or reduced amplitude of pulses, atrophic skin, nail changes and subclavian bruits. In some cases, bruits may be absent due to total vascular occlusion in advanced disease [3]. The initial screening method is a non-invasive Doppler ultrasound of subclavian arteries and is generally preferred due to the low cost [3]. Computed Tomography Angiography (CTA) and Magnetic Resonance Angiography (MRA) are advanced techniques that also help in constructing an anatomic map of the morphology of lesion, location, and length. MRA also provides an additional benefit of determining the flow direction [5]. Despite the use of all the radiologic modalities as above, conventional angiography is still considered the gold standard test for the diagnosis [1].

The concurrent occurrence of carotid artery stenosis in such patients is a rarely reported entity. To date, our patient is the second case of subclavian steal associated with concurrent bilateral carotid artery stenosis. First case of this presentation is mentioned in Aketa 2017, where the patient had an endovascular stent placement following bilateral carotid stenosis with steal phenomenal [6]. Although patients with subclavian steal may be asymptomatic, the reduction in compensatory arterial flow via carotid circulation may precipitate symptoms of steal syndrome [7]. To our surprise, the patient did not show any frontal cerebral symptoms even with severe bilateral carotid stenosis.

There are a variety of treatment options based on the clinical severity. The available treatment options involve medications, endovascular manipulation and surgery [4]. The medical therapy includes aspirin, beta blockers, angiotensin converting enzyme inhibitors and statins. The 2011 AHA/ACC guidelines consider endovascular and surgical approaches to have equivalent efficacy. However, the 2018 European society of Cardiology guidelines has considered percutaneous balloon stenting as the preferred approach due to the 100% success rate with rare significant complications, less invasive nature and need for anesthesia [8,9]. It is recommended to perform progressive intermittent dilations for stent implantation to minimize the complications. Our patient underwent carotid endarterectomy with stenting of occluded vertebral artery which resulted in complete resolution of symptoms. The subclavian artery syndrome is associated with high risk of cardiovascular mortality and cerebrovascular ischemia; therefore, an early diagnosis and prompt treatment is critical for overall management.

## 4 Conclusion

In summary, subclavian steal syndrome is blood flow reversal in vertebral arteries usually caused by severe stenosis of the subclavian artery or innominate artery. The clinical presentation of subclavian steal syndrome is variable, ranging from asymptomatic to cerebrovascular ischemia. Subclavian stenosis is an independent risk factor for overall cardiovascular mortality, and can be readily recognized by duplex ultrasound [5]. Patients with both left subclavian steal syndrome and bilateral carotid artery stenosis require prompt neurosurgical consultation. Clinicians should always have an inclination to rule out carotid stenosis in these patients, as it can be asymptomatic and frequently overlooked. First line treatment includes medical therapy; however, if refractory, carotid endarterectomy should be considered.

## Ethical approval

Obtained.

## Sources of funding

None.

## Author contribution

DS came up with plan, abstract, introduction, conclusion, TS wrote discussion, imagings; BI wrote the case presentation; TA, YS, CA came up with the idea and did final edits.

## Trial registry number

1. Name of the registry: NA
2. Unique Identifying number or registration ID: NA
3. Hyperlink to your specific registration (must be publicly accessible and will be checked): NA

## Guarantor

Talal Almas.

RCSI University of Medicine and Health Sciences.

123 St. Stephen's Green Dublin 2, Ireland.

Talalamas.almas@gmail.com.

+353834212442.

## Consent

Obtained.

## Disclosure

None.

## Declaration of competing interest

None.
